# The Use of Puzzles as a Teaching Technique in Nursing Education: A Randomized Controlled Study

**DOI:** 10.1111/jep.70118

**Published:** 2025-05-22

**Authors:** Nadiye Barış Eren, Bahar Çiftçi

**Affiliations:** ^1^ Department of Fundamental of Nursing Tarsus University Mersin Turkey; ^2^ Department of Fundamental of Nursing Ataturk University Erzurum Turkey; ^3^ HGF Agro, Ata Teknokent Erzurum Turkey

**Keywords:** knowledge level, learning motivation, nursing education, puzzle‐based teaching, self‐efficacy

## Abstract

**Aim:**

This study aimed to examine the influence of puzzle‐based teaching methods on nursing students' levels of knowledge, motivation for learning, and academic self‐efficacy.

**Methods:**

The study employed a randomized controlled pretest‐posttest experimental design conducted between January and June 2024 at Atatürk University's Faculty of Nursing. A total of 70 first‐year nursing students participated, and the intervention and control groups were assigned through random selection. Both groups underwent interactive theoretical instruction on “Excretory Activities” for 2 h across 1 week. However, the intervention group received additional training through puzzle booklets designed by the researchers, while the control group was limited to theoretical instruction. Data collection instruments included the Personal Information Form, Achievement Tests, the Instructional Material Motivation Scale (IMTS), and the Academic Self‐Efficacy Scale (ASES). Data were analyzed using SPSS 22 software.

**Findings:**

The puzzle‐based teaching approach notably improved students' knowledge, motivation, and self‐efficacy. Posttest results for the intervention group showed significant increases in the IMTS sub‐dimensions of “Attention,” “Appropriateness,” “Confidence,” and “Satisfaction” (*p* < 0.05). Additionally, the intervention group achieved higher total IMTS and Knowledge Test scores (β = 0.896, *p* = 0.001) than the control group. Regression analysis indicated that puzzle application substantially and positively affected motivation sub‐dimensions and knowledge levels. The academic self‐efficacy scores of the intervention group were also significantly greater than those of the control group (*p* < 0.05).

**Conclusion:**

Puzzle‐based teaching techniques positively affected knowledge, learning motivation, and academic self‐efficacy. These findings highlight the potential of innovative teaching methods in nursing education, suggesting that puzzle‐based approaches can effectively enhance learning processes and outcomes.

## 1 Introduction

Nursing undergraduate education programs aim to train qualified nurses with the knowledge, skills, and competencies required by contemporary health services. They include both theoretical and practical education [1]. This education process has a structure in which human‐oriented initiatives are integrated with international nursing ethics and values [2]. In this context, it is recommended that different teaching methods and techniques be used to effectively provide students with professional knowledge and skills.

The nursing principles course is one of the most essential courses covering basic nursing skills taught in the first year. In this course, the theoretical foundations and practices of the nursing profession are brought together, and students are prepared to perform professional practices by communicating with sick individuals for the first time in clinical settings. The subject of “Excretory Activities,” which is one of the topics covered in this course, is one of the daily life activities of individuals (Source to be added). It is aimed at nursing students to gain sufficient knowledge and skills in this subject, which includes interventional and complex applications [3]. It is essential to ensure privacy in all nursing practices (Turkish Nurses Association, 2009). Mainly, this subject includes applications where privacy is experienced intensively for both the patient and the nursing student [4]. The literature revealed that students held back during the practice of excretory activities, and this situation negatively affected their skill acquisition ([5]; Selvaraj et al., 2021).

**Figure 1:**
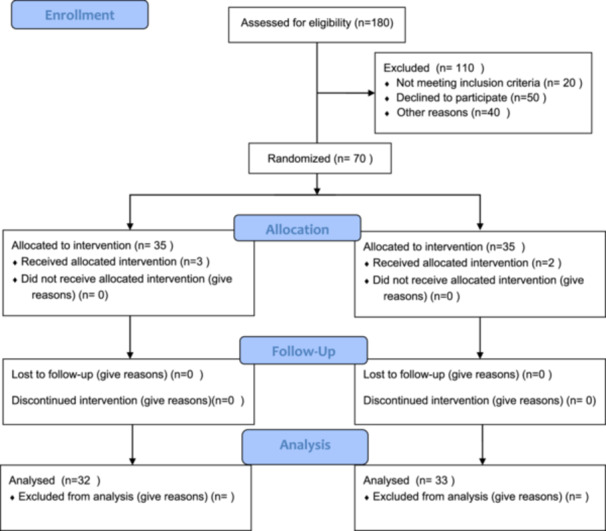
CONSORT 2010 Flow Diagram.

In nursing education, serious games [6], robots [7], virtual reality [8], standard patient [9], web‐based education [10, 11], blended learning [12], flipped classroom [13, 14], simulation [5, 11, 15], scenario‐based learning [16], peer education [17, 18, 19], multimedia‐based education [20], animation and gamification [21], metaverse‐based learning [22] and puzzles [23, 24]. Puzzles, one of these methods, are evaluated by students as a fun, creative, and innovative teaching tool [25]. However, studies on the use of puzzles in education are limited. Despite this, the importance of puzzles as a teaching method is increasing [23, 24, 25, 26, 27]. (Figure 1).

In a study conducted by Zamani et al. [27] with speech and language therapy students, it was found that using puzzles increased the level of knowledge and supported the retention of learning. In addition, it was reported that students' satisfaction levels using the puzzle technique were higher than the control group. Shawahna and Jaber's [24] study on using puzzles in epilepsy treatment also showed increased learning. A study conducted by Köse Tosunöz İ, Deniz Doğan [23] with nursing students showed increased students' knowledge levels. In another study conducted on dentistry students, Qutieshat et al. [26] found that students found the puzzles interesting and meaningful.

Puzzles encourage students to think, and the sense of success they achieve by answering the questions increases their motivation and academic success. Therefore, using the puzzle technique in teaching can provide valuable contributions to nursing education. However, studies on using this technique in nursing education are limited, and there is a significant gap in the literature on this field. In this study, the effect of using puzzles in a course covering nursing practice was evaluated. The puzzles explicitly developed for this field can be used for all nursing students, make learning more fun, and increase participation and motivation in the lessons.

### 1.1 Hypotheses

Using puzzles in nursing education increases the academic success of nursing students.

Using puzzles in nursing education increases the motivation of nursing students.

Using puzzles in nursing education increases the academic self‐efficacy of nursing students.

## 2 Materials Methods

### 2.1 Type of Study

This study used a randomized controlled pretest‐posttest experimental design.

### 2.2 Population and Sample of the Study

The study involved 270 first‐year students across three branches at Atatürk University Faculty of Nursing, with equal distribution based on academic achievement. Each branch uses the same classroom, and subjects are taught by the same lecturer with a consistent methodology. The sample size calculation for this randomized controlled trial (RCT) was conducted using the GPower 3.1.9.7 software. To ensure adequate statistical power, an a priori power analysis was performed. The calculation was based on the primary outcome variable, the difference in knowledge levels between the intervention and control groups, as measured by the Achievement Test scores. The power analysis aimed to achieve a power of 0.80 with a significance level (alpha) of 0.05. An effect size of 0.50 was chosen, representing a medium effect derived from previous studies demonstrating the impact of puzzle‐based teaching techniques on learning outcomes. Additionally, a dropout rate of 5% was considered to account for potential data loss or participant withdrawal during the study period. As a result, the minimum required sample size was calculated as 34 students per group, totaling 68 participants. To increase robustness and account for unforeseen data loss, a final sample size of 70 students was determined. This methodological estimate is crucial to ensuring that the study is sufficiently powered to detect meaningful differences between groups, thereby validating the effectiveness of the intervention.

### 2.3 Inclusion Criteria

The students who participated in the study should be first‐year students, have not received training on ‘excretory activity,’ and have not been absent on the dates.

### 2.4 Exclusion Criteria

Those who could not participate in the intervention group and did not solve the puzzles and students in the control group who reached the puzzle booklet were found to have solved the puzzles.

#### 2.4.1 Randomization and Blinding

In this randomized controlled trial (RCT), the randomization process was conducted using a simple randomization method to ensure unbiased group allocation. Initially, three branches (A, B, and C) consisting of first‐year nursing students were identified as potential groups for the study. To maintain impartiality and prevent selection bias, lots were drawn to assign each branch to either the intervention or control group. The random drawing process was performed under controlled conditions to guarantee transparency and fairness. After the draw, Branch A was assigned as the intervention group, while Branch B was designated as the control group. Branch C was excluded from the study to maintain homogeneity between the selected groups regarding classroom settings and teaching methodology. Furthermore, to eliminate potential biases in outcome assessment, the data analysis was conducted by a statistician blinded to the group assignments. This double‐blind approach was crucial for maintaining the scientific integrity of the study and ensuring objective evaluation of the results. The randomization method and blinding procedures were designed to reduce selection bias and enhance the study's internal validity. Strict precautions were taken to ensure the randomization process's safety and validity. The random drawing was conducted under controlled conditions to minimize manipulation or bias. The process was carefully documented to maintain transparency and reproducibility, safeguarding the study's scientific integrity.

#### 2.4.2 Control of Potential Confounding Factors

We implemented several measures to minimize confounding effects and limit discussion among students. The intervention and control groups were placed in separate classrooms to reduce interaction. Participants were informed that their involvement in the puzzle activity would not affect their exam scores, helping to minimize group effects. After the study, we provided the same puzzle booklet to the control group to ensure fairness and reduce confounding factors.

#### 2.4.3 Data Collection Tools

The data were collected using the “Personal Information Form,” “Achievement Test 1,” “Achievement Test 2,” “Instructional Material Motivation Scale (IMTS),” “Academic Self‐Efficacy Scale (ASES),” and “Puzzle Booklet.”

### 2.5 Personal Information Form

The researchers developed the Personal Information Form based on the literature and included questions about demographic characteristics. The variables recorded in this form included age, gender, family structure, current living arrangement, income status (classified as “Good” if the income level was sufficient to meet daily needs comfortably, and “Bad” if there was difficulty in meeting basic needs), most extended lived place, willingness to choose the department, feeling of belonging to the profession (assessed as “High” if there was a strong sense of commitment and pride in the nursing profession, and “Low” if there was a lack of interest or weak sense of belonging), chronic disease status, enjoyment of playing mobile games, liking puzzles, and playing mobile games for educational purposes. These variables were collected to understand the participants' backgrounds and facilitate subgroup analyzes comprehensively.

### 2.6 Instructional Material Motivation Scale (IMTS)

IMTS was developed by Keller in [28] and adapted into Turkish by Dinçer and Doğanay in [29]. This scale consists of 33 items to measure motivation toward instructional materials across four fundamental dimensions: attention, relevance, confidence, and satisfaction. Each item on the scale is rated using a 5‐point Likert scale, ranging from “Very True” (5) to “Not True” (1). Higher scores indicate greater motivation toward the instructional material. The total possible score on the IMTS ranges from 33 to 165, with higher scores reflecting a higher level of motivation. The overall internal consistency of the scale, as measured by Cronbach's Alpha, is 0.96, indicating high reliability and internal consistency. Each of the four dimensions also demonstrated strong reliability: The Attention dimension consists of 12 items, with a possible score range of 12 to 60. The Cronbach's Alpha for this dimension is 0.89, indicating high internal consistency. This dimension measures the degree to which the instructional material captures and maintains the learner's attention. The Relevance dimension consists of 9 items, with a possible score range of 9 to 45. The Cronbach's Alpha for this dimension is 0.81, reflecting satisfactory internal consistency. This dimension assesses how relevant and applicable the instructional material is to the learner's needs and interests. The Confidence dimension consists of 8 items, with a possible score range of 8 to 40. The Cronbach's Alpha for this dimension is 0.90, demonstrating high reliability. This dimension evaluates how confident learners feel in effectively understanding and using the instructional material. The Satisfaction dimension consists of 4 items, with a possible score range of 4 to 20. The Cronbach's Alpha for this dimension is 0.92, indicating a high internal consistency level. This dimension measures the extent to which learners feel satisfied and content with the instructional experience. The IMTS demonstrates high internal consistency within each dimension, making it reliable for assessing learners' motivation toward instructional materials. The high Cronbach's Alpha values indicate that the scale effectively captures the motivational aspects it aims to measure, thereby supporting the validity of the findings obtained.

### 2.7 “Achievement Test 1” and “Achievement Test 2”

The researchers created multiple‐choice questions after reviewing the literature. Different questions were created for “Achievement Test 1” and “Achievement Test 2” related to the subject of “excretory activities”. Of the 50 multiple‐choice questions considered suitable for the study, 25 were reproduced for each test as many as the number of students and made ready before the application.

Participants in the study were taken to the classroom, where they completed “Achievement Test 1.” The tests were graded on a scale of 100 points. Each correct answer was awarded 4 points to calculate the total achievement scores. The results of both “Achievement Test 1” and “Achievement Test 2” were evaluated using the same scoring system, assigning “4” points for each correct answer and “0” points for each incorrect one. Therefore, a student's highest possible test score was 100, while the lowest was 0. “Achievement Test 1” and “Achievement Test 2” included the topic “Excretory activities”. The topics were prepared with an equal distribution. The achievement tests were not used as students' performance grades or assessment grades. The questions were prepared for the first time and were specific to this study. These questions were not included in the midterm or final exam; this was communicated to the students. Achievement Test 1 Cronbach Alpha value was found to be 0.89. Achievement Test 2 Cronbach Alpha value was found to be 0.78.

### 2.8 “Achievement Test 1” and “Achievement Test 2” Design

Using different questions reduces the learning effect in the pre‐test and posttest and makes the results more objective and reliable. It prevents recall bias that may result from repeating the same questions and more accurately reflects students' actual knowledge growth. Furthermore, using different questions, different subject area dimensions are assessed, increasing content validity and encouraging more in‐depth learning. This method supports meaningful and lasting learning, not just rote learning, and ensures that the assessment process is fair and scientifically based. Assessment Test 1 and Test 2 were developed and implemented by a team of five faculty members, including two associate professors and three professors. The teaching staff members possess expertise in different areas: two associate professors specialize in nursing education, focusing on innovative teaching strategies and curriculum development; two professors are experts in fundamental nursing practices, with extensive experience in clinical applications and patient care; and one professor specializes in measurement and evaluation, significantly contributing to test development and reliability analysis. This diverse academic and clinical expertise ensured that both Assessment Test 1 and Test 2 were designed as reliable and valid measurement tools, accurately assessing theoretical knowledge and practical skills.

### 2.9 Academic Self‐Efficacy Scale (ASES)

Yılmaz et al. adapted the academic self‐efficacy scale into Turkish. This four‐point Likert‐type scale includes seven one‐dimensional items, with scores ranging from 7 to 28, where a higher score indicates greater self‐efficacy. Yılmaz, Gürçay, and Ekici (2007) reported a Cronbach's alpha of 0.79 in their adaptation. In this study, the Cronbach's alpha for academic self‐efficacy was 0.510 in the pretest and 0.605 in the posttest.

#### 2.9.1 Puzzle Booklet

A total of 200 questions on excretory activities were created, which were then organized into puzzles. The https://wordmint.com/puzzles account was used to make the puzzles. Approximately 20 puzzles were created, each containing approximately 10 questions. The puzzles were printed out, and a puzzle booklet was produced. Five teaching staff members evaluated the content and quality of the puzzle booklet, including two associate professors specializing in nursing education, two professors specializing in fundamental nursing practices, and one specializing in measurement and evaluation. Their feedback and recommendations were incorporated to ensure the accuracy and educational value of the puzzles.

#### 2.9.2 Validity and Reliability of Data Collection Tools

Two Achievement Tests used in this study were developed from the literature and validated by ten experts. Revisions were made based on their feedback, and a pilot study with five students evaluated the tools' comprehensibility and usability; these students were not part of the main study. Reliability was assessed using the Spearman‐Brown correlation coefficient, yielding satisfactory results: 0.76 (pretest) and 0.83 (posttest) for Achievement Test 1, and 0.79 (pretest) and 0.85 (posttest) for Achievement Test 2. These values indicate adequate reliability for the instruments.

#### 2.9.3 Data Collection

Data were collected in the Spring semester of the 2023–2024 academic year in the Fundamentals of Nursing course.

*In the first stage*, all students were explained the research, and their consent was obtained. “Personal Information Form” was filled in by the students whose informed consents were obtained and who agreed to participate in the study under observation in the classroom environment. Then, the “Achievement Test 1”, “IMTS,” and “ASES” were administered to all students as a pretest. Fundamentals of Nursing course is one of the compulsory vocational courses in the education of first‐year nursing students, in which basic nursing skill methods are taught. The course consists of 4 h of theoretical, 8 h of clinical, and 2 h of laboratory applications.

*In the second stage*, the subject of excretory activities was taught interactively to all students by the same instructors for 8 h (urinary excretory activity 4 h, intestinal excretory activity 4 h) for 2 weeks. After the theoretical lecture, the instructor demonstrated each skill in the laboratory environment by demonstration method per the checklists for 16 h (urinary excretory activity 8 h, intestinal excretory activity 8 h) for 2 weeks. At the same time, all students were allowed to perform each application individually.

The puzzle booklet previously created for this study was used in the third stage. The researchers made this booklet. During the lesson, the booklet containing 10 puzzle sheets was distributed to the students at the end of each hour and collected from the students at the end of the lesson. In addition, the puzzle booklet was distributed to the students to be repeated later, and all of them were collected at the end of 1 week. The collected booklets were checked, deficiencies and mistakes were noted, and these mistakes were discussed individually with the students, and the correct ones were shown. In addition, the students in the intervention group were warned that their puzzles should not interact with those in the control group.

*In the Control Group (Branch A)*, routine lecturing was continued without any intervention *in the third stage*.

*In the fourth stage*, “Achievement Test 2” was administered to the students in both the intervention and control groups 1 week after the training, and “IMTS” and “ASES” were administered as a posttest.

#### 2.9.4 Data Evaluation

Data were analyzed using SPSS 22. Participant characteristics were presented with percentage and frequency analyzes. A chi‐square analysis assessed the homogeneity of the experimental and control groups regarding sociodemographic characteristics. Normality was evaluated using Skewness and Kurtosis, confirming a normal distribution. Mean scores and standard deviations summarized the scales. Differences in dependent mean scores were analyzed with the “t‐test for Dependent Groups,” differences in independent mean scores were assessed using the “t‐test for Independent Groups,” with effect sizes calculated using Cohen's d. Effect size values were categorized based on Cohen's (1988) guidelines, with 0.2 considered a small effect size, 0.5 a moderate effect size, and 0.8 a large effect size. Regression analysis evaluated the impact of the puzzle application on mean scores, with a significance level set at 0.05. Multiple linear regression examined the relationship between the independent variables and the primary outcome. The assumptions of normality, linearity, homoscedasticity, and multicollinearity were tested and met before analysis. Regression coefficients, confidence intervals, and p‐values were calculated to determine the strength and significance of the associations. Adjusted R‐squared values were reported to indicate the proportion of variance explained by the model.

#### 2.9.5 Ethical Principles of Research

Before starting the study, we obtained approval from the Ethics Committee and written permission from the university to conduct the research. In collecting data, we ensured “Informed Consent” by informing participants about the study, respecting their autonomy by emphasizing voluntary participation, and maintaining confidentiality regarding their information. Both written and verbal consent were collected from willing participants, and we adhered to the Helsinki Declaration of Human Rights to protect individual rights throughout the study.

It was stated that the research results would not affect any performance evaluation, midterm, or final grades and that the control group would be given the puzzle application and the booklet the same way after the research was completed. To avoid risking the reliability of the study and not interacting with the control group, a “Confidentiality Agreement” was signed with the students in the intervention group, which was valid until the research was concluded. At the end of the study, all students in the control group also received the Puzzle booklet to avoid ethical problems.

## 3 Findings

In Group 1, 53.1% of the students were aged 20 or older, 68.8% were female, 71.9% had nuclear families, and 87.5% lived in dormitories. While 65.6% reported low income, 53.1% chose their department willingly, and 71.9% felt they belonged to the profession. Additionally, 78.1% had no chronic illnesses, 68.8% liked mobile games, and 50% used mobile games for education. In Group 2, 60.6% of the students were aged 20 or older, 84.8% were female, 90.9% had nuclear families, and 69.7% lived in dormitories. While 78.8% reported good income, 54.5% willingly chose their department, and 72.7% felt a professional affiliation. Furthermore, 93.9% had no chronic illnesses, 57.6% disliked mobile games, and 57.65% did not use mobile games for education. Chi‐square analysis revealed that the groups differed in terms of “family structure,” “current residence,” “income level,” and “interest in mobile games.” However, they were similar in age, gender, most extended place of residence, voluntary department selection, sense of belonging, chronic illness, and puzzle or mobile game preferences (Table 1).

**Table 1:** Distribution and comparison of descriptive characteristics of students in the experimental and control groups (*n* = 65).

		Groups				
Variables		Experiment (*n* = 32)		Control (*n* = 33)		Test value and significance
		*n*	%	*n*	(%)	
Age	18–19 years old	15	46.9	13	39.4	x^2^ = 0.371 *p* = 0.543^a^
	20 years and over	17	53.1	20	60.6	
Gender	Male	10	31.3	5	15.2	x^2^ = 2.372 *p* = 0.124^a^
	Woman	22	68.8	28	84.8	
Family structure	Core	23	71.9	30	90.9	**x** ^**2**^ = **3.910** ***p*** = **0.048** a
	Wide	9	28.1	3	9.1	
Where he lives now	Family home	4	12.5	10	30.3	**x** ^**2**^ = **4.447** ***p*** = **0.035** a
	Dormitory	28	87.5	23	69.7	
Income Status	Good	11	34.4	26	78.8	**x** ^**2**^ = **13.069** ***p*** = **0.001** a
	Bad	21	65.6	7	21.2	
Longest lived in	Province	13	40.6	22	66.7	x^2^ = 4.753 *p* = 0.093^a^
	District	10	31.3	7	21.2	
	Village	9	28.1	4	12.1	
The status of choosing the department willingly	Yes	15	46.9	18	54.5	x^2^ = 0.382 *p* = 0.536^a^
	No.	17	53.1	15	45.5	
Feeling of belonging to the profession	Yes	23	71.9	24	72.7	x^2^ = 0.006 *p* = 0.939^a^
	No.	9	28.1	9	27.3	
Chronic disease status	Yes	7	21.9	2	6.1	*p* = 0.082^b^
	No.	25	78.1	31	93.9	
Enjoyment of playing mobile games	Yes	22	68.8	14	42.4	**x** ^**2**^ = **4.557** ***p*** = **0.033** a
	No.	10	31.3	19	57.6	
Puzzle liking status	Yes	10	31.3	5	15.2	x^2^ = 2.372 *p* = 0.124^a^
	No.	22	68.8	28	84.8	
Playing mobile games for education	Yes	16	50.0	14	42.4	x^2^ = 0.375 *p* = 0.540^a^
	No.	16	50.0	19	57.6	

*Note:* Bold values indicate statistically significant results (*p* < 0.05).

^a^ = Pearson Chi‐Square Test = Fisher Exact Test.

When comparing the groups, no statistically significant differences were observed in the pretest mean scores for the “Attention,” “Appropriateness,” “Confidence,” and “Satisfaction” subdimensions, as well as the “Motivation Scale for Instructional Material,” “Academic Self‐Efficacy Scale,” and “Knowledge Test” (*p* > 0.05). However, the posttest mean scores for the experimental group were significantly higher than those of the control group across all these measures (*p* < 0.05) (Table 2).

**Table 2:** Comparison of in‐group and between‐group score averages of students in the experimental and control groups (*n* = 65).

		Groups		Intergroup test value and significance^x^
		Experiment (*n* = 32)	Control (*n* = 33)	
		X ± SD	X ± SD	
Caution*	**Pre‐test**	30.84 ± 10.54	31.21 ± 9.52	t = −0.148, *p* = 0.883, ES = 0.037
	**Final test**	39.19 ± 7.55	34.64 ± 8.93	**t** = **2.216, *p*** = **0.030, ES** = **0.550**
In‐group test value and significance^y^		**t** = **−3.281, *p*** = **0.003** **ES** = **0.580**	t = −1.457, *p* = 0.155 ES = 0.254	
Compliance*	**Pre‐test**	25.03 ± 8.26	25.97 ± 7.76	t = −0.472, *p* = 0.638, ES = 0.117
	**Final test**	31.81 ± 5.80	27.76 ± 6.62	**t** = **2.623, *p*** = **0.011, ES** = **0.651**
In‐group test value and significance^y^		**t** = **−3.409, *p*** = **0.002** **ES** = **0.603**	t = −0.916, *p* = 0.366 ES = 0.160	
Trust*	**Pre‐test**	26.53 ± 7.89	27.73 ± 8.06	t = −0.604, *p* = 0.548, ES = 0.150
	**Final test**	34.00 ± 6.12	30.61 ± 7.42	**t** = **2.009, *p*** = **0.049, ES** = **0.499**
In‐group test value and significance^y^		**t** = **−3.816, *p*** = **0.001** **ES** = **0.675**	t = −1.429, *p* = 0.163 ES = 0.249	
Satisfaction*	**Pre‐test**	15.03 ± 5.46	15.52 ± 4.82	t = −0.379, *p* = 0.706, ES = 0.094
	**Final test**	23.84 ± 4.47	20.58 ± 4.95	**t** = **2.792, *p*** = **0.007, ES** = **0.693**
In‐group test value and significance^y^		**t** = **−6.519, *p*** = **0.001** **ES** = **−1.152**	**t** = **−4.051, *p*** = **0.001** **ES** = **0.705**	
Motivation for instructional material scale****	**Pre‐test**	100.53 ± 32.80	103.64 ± 30.35	t = −0.396, *p* = 0.693, ES = 0.098
	**Final test**	128.84 ± 23.29	113.58 ± 27.37	**t** = **2.419, *p*** = **0.018, ES** = **0.600**
In‐group test value and significance^y^		**t** = **−3.583, *p*** = **0.001** **ES** = **0.633**	t = −1.313, *p* = 0.198 ES = 0.573	
Academic self‐efficacy scale	**Pre‐test**	19.56 ± 4.10	19.24 ± 5.61	t = 0.262, *p* = 0.794, ES = 0.065
	**Final test**	21.88 ± 2.49	19.52 ± 4.13	**t** = **2.800, *p*** = **0.007, ES** = **0.690**
In‐group test value and significance^y^		**t** = **−2.886, *p*** = **0.007** **ES** = **0.510**	t = −0.227, *p* = 0.822 ES = 0.381	
Knowledge test	**Pre‐test**	11.38 ± 9.32	15.03 ± 11.40	t = −1.413, *p* = 0.163, ES = 0.350
	**Final test**	82.32 ± 7.78	49.70 ± 8.60	**t** = **15.875, *p*** = **0.001, ES** = **3.971**
In‐group test value and significance^y^		**t** = **−34.886, *p*** = **0.001** **ES** = **6.266**	**t** = **−15.244, *p*** = **0.001** **ES** = **−2.654**	

*Note:* * “Caution,” “Compliance,” “Trust,” and “Satisfaction” are subscales of the Motivation for Instructional Material Scale (IMMS). Academic Self‐Efficacy Scale and Knowledge Test are separate measurement tools x: Independent Groups t test; y: Dependent Groups t Test; ES: Effect Size Cohen's d. Bold values indicate statistically significant results (*p* < 0.05).

In the experimental group, the “Attention” sub‐dimension scores significantly improved from a pre‐test mean of 30.84 ± 10.54 to a posttest mean of 39.19 ± 7.55 (t = −3.281, *p* = 0.003, d = 0.580). In contrast, the control group's scores increased slightly from 31.21 ± 9.52 to 34.64 ± 8.93 but were not statistically significant (t = −1.457, *p* = 0.155, d = 0.254). For the “Appropriateness” sub‐dimension, the experimental group's mean rose from 25.03 ± 8.26 to 31.81 ± 5.80, significantly improving (t = −3.409, *p* = 0.002, d = 0.603). The control group's mean increased marginally from 25.97 ± 7.76 to 27.76 ± 6.62 without statistical significance (t = −0.916, *p* = 0.366, d = 0.160). In the “Trust” sub‐dimension, the experimental group's scores significantly improved from 26.53 ± 7.89 to 34.00 ± 6.12 (t = −3.816, *p* = 0.001, d = 0.675), while the control group showed a nonsignificant increase from 27.73 ± 8.06 to 30.61 ± 7.42 (t = −1.429, *p* = 0.163, d = 0.249). The “Satisfaction” sub‐dimension also significantly increased in the experimental group from 15.03 ± 5.46 to 23.84 ± 4.47 (t = −6.519, *p* = 0.001, d = 1.152). Similarly, the control group improved from 15.52 ± 4.82 to 20.58 ± 4.95, with the difference being statistically significant (t = −4.051, *p* = 0.001, d = 0.705).

The experimental group's mean score on the “Motivation Scale for Instructional Materials” significantly improved from 100.53 ± 32.80 to 128.84 ± 23.29 (t = −3.583, *p* = 0.001, d = 0.633). In contrast, the control group's mean score increased slightly from 103.64 ± 30.35 to 113.58 ± 27.37, but this change was not statistically significant (t = −1.313, *p* = 0.198, d = 0.573).

The experimental group's “Academic Self‐Efficacy Scale” mean score significantly increased from 19.56 ± 4.10 to 21.88 ± 2.49 (t = −2.886, *p* = 0.007, d = 0.510). In contrast, the control group's mean score rose slightly from 19.24 ± 5.61 to 19.52 ± 4.13, but this change was not statistically significant (t = −0.227, *p* = 0.822, d = 0.381).

The experimental group's mean “Knowledge Test” score significantly increased from 11.38 ± 9.32 to 82.32 ± 7.78 (t = −34.886, *p* = 0.001, d = 6.266). Similarly, the control group significantly increased from 15.03 ± 11.40 to 49.70 ± 8.60 (t = −15.244, *p* = 0.001, d = 2.654).

Table 3 shows the results of the “Regression Analysis with Representative Variable,” which identified seven statistically significant models (*p* < 0.05). The analysis revealed that the puzzle application had a significant impact on the “Attention Subdimension” (β = 0.269, *p* = 0.030), “Appropriateness Subdimension” (β = 0.314, *p* = 0.011), “Confidence Subdimension” (β = 0.245, *p* = 0.049), “Satisfaction Subdimension” (β = 0.332, *p* = 0.007), “Motivation Scale for Instructional Material” (β = 0.291, *p* = 0.018), “Academic Self‐Efficacy Scale” (β = 0.331, *p* = 0.007), and “Knowledge Test” (β = 0.896, *p* = 0.001).

**Table 3:** Regression analysis results regarding the effect of puzzle implementation on score averages.

Dependent variable	Model	Variables	B	S. Error	β	*t*	*p*
Caution*	1	Fixed	34.636	1.441		24.032	**0.001**
		Puzzle Application ‐ Experiment	4.551	2.054	0.269	2.216	**0.030**
	**R** = **0.269, R** ^**2**^ = **0.072, F** = **4.909, p** = **0.030**						
Compliance*	1	Fixed	27.758	1.085		25.586	**0.001**
		Puzzle Application ‐ Experiment	4.055	1.546	0.314	2.623	**0.011**
	**R** = **0.314, R** ^**2**^ = **0.098, F** = **6.878, *p*** = **0.011**						
Trust*	1	Fixed	30.606	1.185		25.826	**0.001**
		Puzzle Application ‐ Experiment	3.394	1.689	0.245	2.009	**0.049**
	**R** = **0.245, R** ^**2**^ = **0.060, F** = **4.038, *p*** = **0.049**						
Satisfaction*	1	Fixed	20.576	0.821		25.053	**0.001**
		Puzzle Application ‐ Experiment	3.268	1.171	0.332	2.792	**0.007**
	**R** = **0.332, R** ^**2**^ = **0.110, F** = **7.795, *p*** = **0.007**						
Motivation scale for instructional material ****	1	Fixed	113.576	4.429		25.641	**0.001**
		Puzzle Application ‐ Experiment	15.268	6.313	0.291	2.419	**0.018**
	**R** = **0.291, R** ^**2**^ = **0.085, F** = **5.849, *p*** = **0.018**						
Academic self‐efficacy scale	1	Fixed	19.515	0.596		32.760	**0.001**
		Puzzle Application ‐ Experiment	2.360	0.849	0.331	2.780	**0.007**
	**R** = **0.331, R** ^**2**^ = **0.109, F** = **7.726, *p*** = **0.007**						
Knowledge test	1	Fixed	49.697	1.430		34.745	**0.001**
		Puzzle Application ‐ Experiment	32.626	2.055	0.896	15.875	**0.001**
	**R** = **0.896, R** ^**2**^ = **0.803, F** = **252.018, *p*** = **0.001**						

*Note:* * “Caution,” “Compliance,” “Trust,” and “Satisfaction” are subscales of the Motivation for Instructional Material Scale (IMMS). Academic Self‐Efficacy Scale and Knowledge Test are separate measurement tools. Bold values indicate statistically significant results (*p* < 0.05).

## 4 Discussion

This study evaluated the effect of puzzle‐based teaching techniques on nursing students. It was determined that the knowledge level of the students in the experimental group increased more than in the control group. It was observed that the experimental group could maintain the knowledge without forgetting and more effectively participate in the learning process. The literature frequently emphasizes the contribution of puzzle and game‐based teaching techniques to the learning process. In particular, it has been reported that these techniques increase the knowledge level, ensure information retention, and support the development of psychomotor skills. Demir Acar and Çaylak Altun [30] stated that puzzle‐based teaching techniques increased students' knowledge levels, and visual learning style played an essential role in this process. Similarly, Zamani et al. [27] stated that using puzzles increased students' participation in learning and ensured learning retention. Qutieshat et al. [26] stated that puzzles are considered an interesting and success‐oriented tool, and Shawahna and Jaber [24] showed that puzzles are effective in learning complex subjects. Aydin and Ince [31] found that these techniques support the development of psychomotor skills. Köse Tosunöz İ, Deniz Doğan [23] emphasized that the use of puzzles increased the knowledge levels of the experimental group students to a higher level than those in the control group, and Torres et al. [25] stated that puzzles increased overall achievement. Meta‐analysis studies also support the positive effects of puzzle‐based techniques. Ozkan and Uslusoy [32] showed that puzzle techniques positively affected students' academic achievement, skills, and attitudes. However, Sumanasekera et al. [33] reported that puzzles helped students remember concepts but did not significantly affect knowledge scores. This shows the effect of individual differences in the learning process. In conclusion, puzzle‐based teaching techniques are an effective method that increases knowledge retention and learning motivation. Using innovative techniques in applied disciplines, such as nursing education, strengthens the learning process and increases students' commitment to the course.

Game‐based learning strategies are practical tools for increasing student motivation, facilitating understanding of complex topics, and developing critical thinking. The findings of this study showed that game‐based learning effectively improved self‐efficacy levels. This is consistent with Bandura's (1997) self‐efficacy theory and supports the positive effect of individuals' success experiences on self‐efficacy. Uz Bilgin et al. [34] emphasized the positive impact of debriefing strategies used in game‐based learning on self‐efficacy. Elviyasmi et al. [35] and Li [36] stated that puzzle‐based learning positively affects students' cognitive processes and provides more effective learning of concepts. Sadati et al. [37] showed that online puzzle‐based teaching provided lower learning outcomes than interactive online course methods. Ajayi and Ogbeba [38] stated that the three‐dimensional puzzle‐based strategy increased student engagement and achievement. Hanukov and Smolyanova [39] revealed that these strategies positively affected learning outcomes by encouraging collaboration and collective learning. Shepherd [40] and Khorammakan et al. [41] reported that such methods contribute to students' understanding of complex concepts and improve their performance in clinical practice. The literature generally supports the idea that game‐based learning has strong and positive effects on student motivation, self‐efficacy, and learning outcomes.

Innovative techniques in educational processes increase students' motivation to learn and strengthen their participation in lessons. Studies have supported the idea that using puzzles increases students' motivation. Zamani et al. [27] reported that puzzles increased the retention of information, enabled students to participate more actively in the learning process, and increased their overall satisfaction levels. Similarly, Qutieshat et al. [26] stated that puzzles are an engaging tool that supports learning. In addition, Shawahna and Jaber [24] showed that using puzzles effectively teaches complex subjects and enables students to remember information for extended periods. Sadati et al. [37] compared online puzzle‐based instruction with interactive lecture methods and found that interactive lecture methods provided higher learning outcomes. Ajayi and Ogbeba [38] stated that the three‐dimensional puzzle‐based strategy increased student motivation and significantly improved learning outcomes. Torres et al. [25] noted that using puzzles increased student achievement by increasing knowledge retention. Ozkan and Uslusoy [32] emphasized the positive effects of puzzle‐based techniques on academic achievement and skills in their meta‐analysis study. However, Aras, Ciftci [42] showed that the effect of other methods (such as question‐answer and Kahoot) on motivation was not as strong as puzzle‐based techniques. These findings suggest that puzzle‐based teaching techniques are used not only as a learning tool but also as an effective method that makes the process more fun and meaningful. students' active participation while solving puzzles is a key factor that increases motivation by making the learning process a fun experience [27].

Motivation for the instructional material is an essential factor that increases students' participation in learning and enables them to establish a meaningful connection with the material. Puzzle‐based techniques increase motivation towards the material, make students find the material more interesting, and take a more active part in the learning process. This study found that puzzle application created a strong interaction between attention, relevance, confidence, satisfaction, motivation, academic self‐efficacy, and knowledge levels. A positive relationship was found between these dimensions, and an increase in one positively affected the others. For example, increased attention increased students' interest in the material, which led to a more appropriate perception of the material. Zamani et al. [27] also stated that puzzles make learning processes more effective by increasing students' attention levels. The increase in the perception of appropriateness led to an increase in satisfaction. Students felt more satisfaction when they found the material meaningful and appropriate. This increased engagement and motivation in the learning process. Fatima et al. [43] stated that using meaningful materials increased students' motivation and satisfaction. The increase in the sense of confidence reinforced the students' belief in the learning process and enabled them to participate more actively. This directly strengthened the perception of academic self‐efficacy. Shawahna and Jaber [24] reported that puzzle‐based techniques supported the learning process by giving students confidence. Increasing motivation and satisfaction levels strengthened students' commitment to the learning process. Increased motivation increased interest in the learning material and retention of information. Torres et al. [25] stated that increased satisfaction levels negatively affected learning processes. This engagement contributed to students' remembering information for longer and having more effective learning experiences. Academic self‐efficacy reflects students' confidence in themselves in learning processes. The self‐efficacy‐enhancing effect of the puzzle application enabled students to participate in learning processes more confidently and actively. When students accessed information through puzzles and produced correct solutions, their self‐efficacy perceptions were strengthened. Shawahna and Jaber [24] stated that such techniques contribute positively to learning by increasing self‐confidence. The increased self‐efficacy increased the commitment to the learning material and made the learning processes more effective. In conclusion, puzzle‐based teaching techniques positively affected learning by creating a strong link between motivation, academic self‐efficacy, and knowledge level of the teaching material. As motivation increased, it was observed that there was an increase in self‐efficacy perception, which contributed to knowledge acquisition. These findings show that puzzle‐based techniques provide a holistic contribution to learning processes and can be used effectively in different areas of education.

### 4.1 Limitations of the Research

This study was conducted only with first‐year nursing students studying at the nursing faculty of a university. This may limit the generalizability of the findings obtained to students at different educational levels or universities. In addition, the possibility of interaction between the students in the intervention and control groups is considered a significant limitation that may affect the study results. To minimize the interaction between the groups, the intervention and control groups were located in different classes and clearly stated to both groups that the study would not affect their academic assessment grades. However, despite this, possible information sharing between students may not have been completely prevented. To overcome such limitations, it is recommended to include larger sample groups and students from different universities in future studies. In addition, technological solutions or other mechanisms can be used to reduce the risk of interaction between groups further. Evaluating the effects of this method in different contexts with more inclusive and interdisciplinary studies will increase the general validity of the technique.

### 4.2 Conclusion and Recommendations

This study evaluated the effect of puzzle‐based teaching techniques on nursing students. The results showed that the puzzle application significantly and positively affected students' knowledge levels, learning motivation, and academic self‐efficacy. The posttest scores of the students in the experimental group were found to be statistically significantly higher than those of the control group in the knowledge test and motivation scale. Regression analysis findings supported that puzzle‐based techniques positively affected all motivation sub‐dimensions and knowledge levels. It was determined that the puzzle‐based teaching method effectively makes knowledge permanent and improves the learning process, especially in applied disciplines. These techniques increased the interest and motivation in the course by enabling students to participate more actively in the learning process.

In this direction, it is recommended that puzzle‐based teaching techniques be used regularly in applied areas such as nursing education, and various educational materials should support these techniques. Integrating puzzles and other game‐based learning methods into the curriculum of nursing undergraduate programs may increase student motivation and academic success. Repeating this study on nursing students in different universities and educational levels will increase the generalizability of the method. In addition, instructors should be informed about puzzle‐based techniques and integrate them into their courses. In addition, studies evaluating the long‐term effects of puzzle‐based techniques should be conducted, and the effects of these techniques on retention and professional skill development should be examined. These recommendations are essential for disseminating innovative techniques in nursing education and providing more effective learning experiences in the education process.

### 4.3 Clinical Relevance

This study shows that puzzle‐based teaching techniques significantly affect nursing students' knowledge level, learning motivation, and academic self‐efficacy. Using innovative educational methods such as puzzles in teaching complex subjects such as “excretory activities,” which form a strong basis for clinical practice, enables students to participate more actively in the learning process and makes the knowledge permanent. In terms of clinical education, this technique can be considered a tool to improve students' psychomotor skills and clinical decision‐making abilities. In addition, it can increase students' self‐confidence and reduce their stress and anxiety levels. Integrating puzzle‐based teaching techniques with clinical simulations or laboratory applications may make nursing students more prepared and competent in actual patient care. This method also enriches learning experiences by making education fun and interactive.

## Author Contributions

Study conception and design: Nadiye Barış Eren, Bahar Çiftçi. Data collection: Nadiye Barış Eren. Data analysis and interpretation: Nadiye Barış Eren, Bahar Çiftçi. Drafting of the article: Nadiye Barış Eren, Bahar Çiftçi. Manuscript writing: Nadiye Barış Eren, Bahar Çiftçi. Critical revision of the article: Bahar Çiftçi. All listed authors meet the authorship criteria, and all authors agree with the manuscript's content.

## Ethics Statement

Before starting the study, approval was obtained from the Ethics Committee.

## Conflicts of Interest

The authors declare no conflicts of interest.

## Data Availability

The data that supports the findings of this study are available in the supporting material of this article.
